# Age Effects on Neural Discriminability and Monitoring Process During Memory Retrieval for Auditory Words

**DOI:** 10.3389/fnagi.2022.884993

**Published:** 2022-07-19

**Authors:** Xuhao Shao, Wenzhi Liu, Ying Guo, Bi Zhu

**Affiliations:** ^1^State Key Laboratory of Cognitive Neuroscience and Learning, Beijing Normal University, Beijing, China; ^2^Institute of Developmental Psychology, Beijing Normal University, Beijing, China; ^3^Beijing Key Laboratory of Brain Imaging and Connectomics, Beijing Normal University, Beijing, China; ^4^IDG/McGovern Institute for Brain Research, Beijing Normal University, Beijing, China; ^5^School of Education, Cangzhou Normal University, Cangzhou, China

**Keywords:** aging, memory, fMRI, multivoxel pattern classification, auditory

## Abstract

After hearing a list of words (e.g., dream, awake, and bed), older adults tended to have more difficulty than younger adults in distinguishing targets (e.g., dream) from lures (e.g., sleep) and foils (e.g., pen) in a visual recognition test. Age-related reduction in neural discriminability in the visual cortex has been linked to deficits in memory discriminability of pictures. However, no study has examined age differences in auditory discrimination and prefrontal monitoring during true and false memory retrieval after hearing words. The current study used a visual recognition test following an auditory study of words and showed that older adults had lower true recognition and higher propensity for high-confidence false recognition compared to young adults. Using classification-based multivariate pattern analysis for functional neuroimaging data during memory retrieval, we found that neural activation patterns in the primary auditory cortex could be used to distinguish between auditorily-studied targets and unstudied lures in young adults, but not in older adults. Moreover, prefrontal monitoring for lures was weaker in older adults as compared to young adults. Individual differences analysis showed that neural discriminability in the primary auditory cortex was positively related to true recognition, whereas prefrontal activation for lures was negatively related to the propensity for high-confidence false recognition in young adults but not in older adults. Together, age differences in true and false memories following auditory study are associated with reduced neural discriminability in the primary auditory cortex and reduced prefrontal monitoring during retrieval.

## Introduction

Memory retrieval for spoken language deteriorates with age. Along with a decline in true memory for words actually heard, older adults sometimes show increased susceptibility to high-confidence false memory for words not heard (Norman and Schacter, 1997; Schacter et al., 1999; Failes et al., 2020). To examine age differences in true and false memories, the Deese/Roediger-McDermott (DRM) paradigm has been used widely (Roediger and McDermott, 1995; Balota et al., 1999). For example, after hearing a list of words (e.g., “dream,” “awake,” “bed,” and so on), people usually recognize target words like “dream” as old (i.e., true recognition), but they may also recognize unstudied but semantically related lures like “sleep” as old (i.e., false recognition), and they are less likely to recognize unstudied and unrelated foils like “pen” as old (i.e., foil recognition). Compared to young adults, older adults have more difficulty in distinguishing between auditorily studied targets and unstudied lures and foils in the visual recognition test (Kensinger and Schacter, 1999; Smith et al., 2005).

Numerous studies have been conducted in the visual study and visual test condition to explore the neural mechanisms underlying age differences in memory performance and found two potential mechanisms: (1) the neural dedifferentiation of representation in the visual cortex and (2) the retrieval monitoring in the prefrontal cortex (Park et al., 2004; Carp et al., 2011; McDonough et al., 2013, 2014; Devitt and Schacter, 2016; Kirmsse et al., 2018; Trelle et al., 2019). As shown in previous aging studies using the visual learning of DRM words, visuocortical activation decreased with age at encoding for true memory, whereas the left lateral prefrontal activation decreased with age at retrieval for false memory (Dennis et al., 2007, 2008). During the retrieval of visual objects, the ability of the primary visual cortex to discriminate true from false memory decreased with age (Bowman et al., 2019). To date, those works have focused on age deficits in visual memory, while age differences in the neural correlates of auditory true and false memories are unclear. To our knowledge, no study has examined age differences in neural activation and discriminability during retrieval of auditory true and false memories. The purpose of this study was to examine the effect of age on neural discriminability in the sensory cortex and prefrontal monitoring during memory retrieval of auditory true and false memories.

Age-related neural dedifferentiation in the auditory cortex may contribute to the age deficits in distinguishing true and false memories after listening to words. During the perception of auditory stimuli, neural activation patterns in the auditory cortex are less distinctive in older adults than in young adults (Du et al., 2016; Lalwani et al., 2019; Erb et al., 2020). However, age deficits in auditory cortical reactivation specificity may be more severe during memory retrieval than during perception, especially in the primary or low-level auditory cortex (St-Laurent et al., 2014). During memory retrieval for auditory information, the primary auditory cortex (e.g., Heschl's gyrus) can be reactivated (Nyberg et al., 2000; Wheeler et al., 2000). Supporting the sensory reactivation hypothesis, prior neuroimaging studies in young adults reported that large portions of the auditory cortex and nearby regions (e.g., superior temporal gyrus, temporal plane, and supramarginal gyrus) showed greater activation for true than false memories during the visual recognition test after listening to DRM words (Schacter et al., 1996; Abe et al., 2008). During retrieval, true memory activated these temporoparietal regions to a greater degree than false memory, which may reflect greater recollection of auditory details for targets than for lures in young adults (Schacter and Slotnick, 2004; Straube, 2012). However, it remains unclear how aging affects the neural discriminability between heard and unheard words in auditory cortical subregions during memory retrieval. Besides, listened words could induce mental imagery in the visual cortex during encoding (D'Esposito et al., 1997). The reactivation of the mental imagery of listened words in the primary visual cortex may contain sensory details (Kosslyn and Thompson, 2003), which could be used to distinguish between true and false memories. Therefore, one goal of this study is to examine age differences in neural activation patterns in the sensory cortex (i.e., auditory and visual cortex) for distinguishing between auditorily studied targets and unstudied lures and foils in the visual recognition test.

In addition to the dysfunction of the auditory cortex during memory retrieval, impairment of prefrontal monitoring processes may also contribute to age differences in false memory of unheard lures. After visual learning of DRM words, the left lateral prefrontal cortex showed greater monitoring process activation for lures than foils in young adults (Ye et al., 2016; Zhu et al., 2019) and exhibited age-related decreases in activation during high-confidence false memory retrieval (Dennis et al., 2008). According to the source monitoring framework, this age-related activation decrease in the left lateral prefrontal cortex may reflect a reduced monitoring process for lures during memory retrieval (Johnson et al., 1993; Devitt and Schacter, 2016; Fandakova et al., 2018). As shown in previous behavioral studies, monitoring deficits have been linked to an increased propensity to make high-confidence false memory in older adults (Shing et al., 2009; Fandakova et al., 2013). However, it remains unclear whether the left lateral prefrontal cortex is implicated in age differences in false memory retrieval after listening to words. Thus, another goal of the current study was to investigate whether older adults had reduced prefrontal monitoring for lures in the visual recognition test following auditory learning compared with young adults.

In the current study, after listening to a series of DRM word lists, young and older participants were asked to complete a visual recognition test in which memory judgments were made for three types of words (i.e., targets, similar lures, and novel foils) in the functional magnetic resonance imaging (fMRI) scanner. This study (1) investigated age differences in neural discriminability in the sensory cortex using a classification-based multivoxel pattern analysis (MVPA), and (2) examined age differences in the prefrontal monitoring process using univariate activation analysis for imaging data during memory retrieval. To investigate the neural discriminability in the sensory cortex, we selected four regions of interest (ROIs), including the primary and secondary regions in the auditory and visual cortex. Since the primary auditory/visual cortex is assumed to support reactivations of details of listened words, we predicted that the neural activation patterns in the primary auditory/visual cortex could be used to differentiate between targets and lures in young adults. Furthermore, older adults should have more difficulty distinguishing targets and lures from activation patterns in the primary auditory/visual cortex compared to young adults. To investigate the age difference in the prefrontal monitoring process during retrieval, we compared neural activation in the left lateral prefrontal cortex in response to lures in young and older adults. We predicted that older adults should have lower activation to lures in the left lateral prefrontal cortex compared to young adults. In addition, individual differences analysis was used to explore neural-behavioral correlations in young and older adults.

## Materials and Methods

### Participants

Twenty-seven young adults (18 women and 9 men, mean age: 23 ± 2 years old, ranging from 19 to 26 years) and 27 older adults (15 women and 12 men, mean age: 65 ± 5 years old, ranging from 54 to 74 years) were included in the final analysis. This sample size was predetermined by following our previous studies using the same experimental design (Ye et al., 2016; Zhu et al., 2019). Seven additional older adults were excluded from this study before data analysis because they were unable to understand the task requirements and gave up during the fMRI scan. Young adults were students enrolled from Beijing Normal University and older adults were enrolled from the nearby communities. All participants were right-handed, had normal vision and hearing, and had no history of psychiatric or neurological diseases. Older adults were screened for depression using the Beck Depression Inventory–II (BDI-II) (Beck et al., 1996) and screened for dementia using the Mini–Mental State Exam (MMSE) (Folstein et al., 1975). The means and standard deviations for BDI-II and MMSE scores and educational years in older adults were 7 ± 6, 29 ± 1, and 12 ± 3, respectively. Written consent was obtained from each participant. The current study was approved by the Institutional Review Board of the State Key Laboratory of Cognitive Neuroscience and Learning at Beijing Normal University, China.

### Materials

Nine lists of 12 two-character Chinese words were used. Each list describes one theme (e.g., “dream,” “awake,” “bed,” “doze,” “yawn,” “snore,” “drowsy,” “blanket,” “sleep,” “rest,” “tired,” and “pillow”). These materials were translated and adapted from materials used in Roediger and McDermott (1995) and have been used in our recent fMRI studies (Ye et al., 2016; Zhu et al., 2019). For each list, eight words would be studied (e.g., “dream,” “awake,” “bed,” “doze,” “yawn,” “snore,” “drowsy,” and “blanket”). In the recognition test, for each list, four of the eight studied words would be used as targets (e.g., “dream,” “bed,” “yawn,” and “drowsy”), while four semantically related but unstudied words would be used as lures (e.g., “sleep,” “rest,” “tired,” and “pillow”). Moreover, 36 semantically unrelated and unstudied two-character Chinese words were used as foils (e.g., “pen,” “recycle,” “factory,” and “orange”) in the recognition test. Study items were presented auditorily in a female voice and test items were presented visually in the center of the computer screen.

### Experimental Design

**Figure 1:**
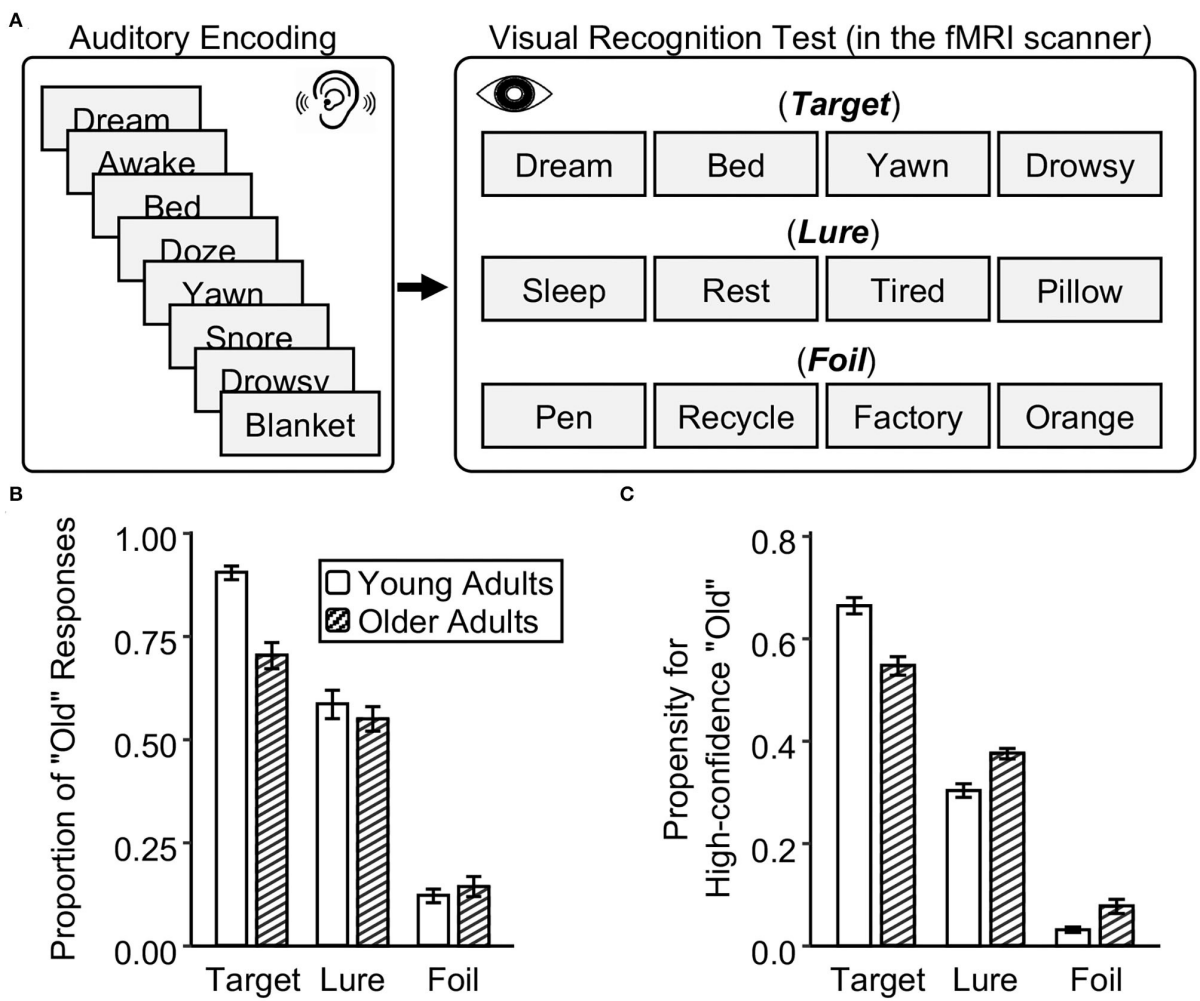
Experimental design and memory performance in young and older adults. **(A)** After listening to words, young and older adults were asked to make memory judgments on studied targets, similar lures, and novel foils in a visual recognition test in the fMRI scanner. **(B)** The proportion of “old” responses in the recognition test. **(C)** The propensity for high-confidence recognition. The error bar indicates the standard error of the means.

During auditory encoding, participants heard 9 lists of 8 words (i.e., 72 words in total), and they were asked to memorize each word (Figure 1A). There was a 3-s visual cue (e.g., “List 1”) before the start of each word list. Each trial started with a 0.5-s fixation point, followed by an auditorily presented Chinese word for 1 s. All words used in the current study are composed of two characters in Chinese, so they can be pronounced with the same duration of one second. To help participants to remember these words, participants were asked to make a pleasantness judgment on each word as quickly and accurately as possible, by pressing 1 of 4 buttons (1 = “very pleasant,” 2 = “mildly pleasant,” 3 = “mildly unpleasant,” 4 = “very unpleasant”) within 2 s. Each word was presented only once. The order of presentation for 8 studied words in a list was based on the level of semantic associations between studied words within each list (i.e., the word with the highest semantic association level showed first). After studying all 9 lists, there would be a 10-min interval before the recognition test (i.e., the preparation for the scanning, such as the safety check).

During visual retrieval (inside the fMRI scanner), a slow event-related design (12 s for each trial) was used to obtain better estimates of single-trial blood-oxygen-level-dependent (BOLD) response. Since BOLD signals in the slow-event design are less affected by the temporal overlap for adjacent trials (Mumford et al., 2012; Choupan et al., 2020). This procedure has been well-validated (Liu, 2012; Gordon et al., 2014; Zheng et al., 2021). Each trial started with a 1-s fixation point, followed by a visually presented Chinese word for 3 s. Participants were asked to judge whether they had studied the word earlier by pressing 1 of 4 buttons with their left or right index finger or middle finger (1 = “definitely new,” 2 = “probably new,” 3 = “probably old,” 4 = “definitely old”) within 3 s. The use of left vs. right hand for new vs. old responses was counterbalanced across participants. Next, participants were asked to complete a self-paced perceptual judgment task for 8 s to prevent participants from further processing these words. An arrow image pointing to the left or the right was randomly presented in the center of the screen. Participants were asked to identify the orientation of the arrow as quickly and accurately as possible by pressing 1 of 2 buttons (1 = “left,” 4 = “right”). In total, 108 words (i.e., 36 targets, 36 lures, and 36 foils) were presented over three (scanning) runs, and the order of presentation for these words was pseudorandomized. To test whether the perceptual judgment task is an appropriate baseline for the memory task as reported by Stark and Squire (2001), we conducted univariate analyses for the contrast of the memory task and perceptual judgment task in the whole brain for both the age groups. We found that several brain regions, including the sensory cortex, frontal lobe, and parietal lobe, showed higher activation for memory tasks than perceptual judgment tasks. It suggested that using the perceptual judgment task is an appropriate baseline for memory tasks in the current study (see more details in Supplementary Figure 1).

### Behavioral Analysis

True recognition (i.e., target judged as old), false recognition (i.e., lure judged as old), and foil recognition (i.e., foil judged as old) were calculated by the proportions of “old” response (scored as 3 or 4) for targets, lures, and foils in young and older adults, separately. In addition, following the methods of previous aging studies using similar experimental designs (Dennis et al., 2007, 2008), we calculated the propensity for high-confidence recognition (scored as 4) for each item type (i.e., target, lure, and foil) in young and older adults, separately. As shown in previous studies, the index of the propensity for high-confidence recognition was used to understand the age differences in judging a certain type of item as definitely learned. For example, the propensity for high-confidence false recognition was calculated by dividing the number of “definitely old” responses to lure by the total number of “definitely old” responses to all three types of items. Among 108 words in the recognition test (i.e., 36 targets, 36 lures, and 36 foils), if a participant judged 30 targets, 15 lures, and 2 foils as “definitely old,” then the propensity for high-confidence false recognition would be 0.38 (i.e., 15/(30+15+2) = 0.38). The same logic was used to calculate these indices for targets and foils, respectively. For each age group, we used paired *t-*tests to compare the recognition performance between targets, lures, and foils. For each item type, we used independent *t-*tests to compare age differences in the proportion of “old” responses and the propensity of high-confidence recognition. The reaction time during the recognition test was calculated for target judged as old, lure judged as old, lure judged as new, and foil judged as new. The mixed-design ANOVA with age groups (young and old) as a between-subjects variable and response types (target judged as old, lure judged as old, lure judged as new, and foil judged as new) as a within-subjects variable was conducted to examine the interaction between age group and response type on the reaction time.

### fMRI Data Collection and Preprocessing

A 3.0 T Siemens Magnetom Trio scanner at Beijing Normal University Brain Imaging Center was used for brain imaging scans. The following functional imaging acquisition parameters were used for a single-shot T2^*^-weighted gradient-echo, EPI sequence: TR/TE/θ = 2,000 ms/25 ms/90°, FOV = 192 × 192 mm, matrix = 64 × 64, and slice thickness 3.0 mm. To cover the whole cerebrum and partial cerebellum, 41 contiguous axial slices parallel to the AC-PC line were obtained. The following structural MRI parameters was used for a T1-weighted, 3D, gradient-echo pulse-sequence (MPRAGE): T1/TR/TE/θ= 1,100 ms/2,530 ms/3.39 ms/7°, FOV = 256 × 256 mm, matrix = 256 × 256, and slice thickness = 1.33 mm. A total of 144 sagittal slices were acquired to provide high-resolution structural images of the whole brain.

Data preprocessing was conducted using the pipeline fMRIPrep v1.4.0 (Esteban et al., 2019). The T1-weighted images were corrected for intensity non-uniformity using N4BiasFieldCorrection and skull-stripped using the OASIS_30_ANTs template (antsBrainExtraction.sh). Spatial normalization was performed with the MNI152NLin2009cAsym template through non-linear registration with antsRegistration (ANTs). Functional data were coregistrated to the corresponding structural image by boundary-based registration with 9 degrees of freedom in using the bbregister (FreeSurfer). Then, the slice-timing correction using 3dTshift (AFNI) and motion correction using MCFLIRT (FSL) was performed. Using antsApplyTransforms (ANTs) with Lanczos interpolation, the motion-correcting transformations, functional-to-structural transformation, and structural-to-template warp were concatenated and applied in a single step. The BOLD time series were resampled to the 2 × 2 × 2 mm resolution in the native space and the standard space (MNI152NLin2009cAsym template). The native space was used for the ROI analysis of pattern classification. The standard space was used for the whole-brain analysis of pattern classification and univariate activation. Using the implementation of Nipype, the frame-wise displacement was calculated for each functional run. Then, using a non-linear high-pass filter with a 100-s cut-off, they were filtered temporally. Smoothed data with a 5-mm FWHM Gaussian kernel was used for the whole-brain analysis of univariate activation. Unsmoothed data were used for both ROI analysis and whole-brain searchlight analysis of pattern classification.

### Single-Trial Estimation

The General Linear Model (GLM) as implemented in FSL v5.0.9 was used to model the data. The GLM was used to compute the *t* map for each of the 108 words in the recognition test for young and older adults, separately. The presentation of each stimulus was modeled as an impulse in this single-trial model, and it convolved with a canonical hemodynamic response function (double gamma). To obtain reliable estimates of single-trial responses, the least-square separate method was used (Mumford et al., 2012). The GLM also included nuisance regressors for six motion parameters, framewise displacement (FD), and reaction time. The *t* value for each stimulus of each participant was used to calculate the neural classification accuracy, which was more reliable compared with the beta value (Walther et al., 2016).

### Neural Pattern Classification Analysis

To examine in which brain region the neural patterns of targets are distinguishable from lures and foils during the recognition in young or older adults, we used two binary classifications and calculated the classification accuracies for discriminating targets from lures and discriminating targets from foils, by including all trials regardless of response accuracy. The reason we use two binary classifications instead of a three-way classification is that there is a possibility that foils can be distinguished from targets and lures, but the latter two cannot be distinguished from each other. For each ROI, we used the linear support vector machines classifier from the scikit-learn package (v0.21.3) in Python for classification. We used the standard leave-one-run-out cross-validation procedure, with two of three runs used for training and the remaining one run used for testing, resulting in a total of three validations. Classifiers' accuracies across validations were averaged to form a final output accuracy for each participant.

**Figure 2:**
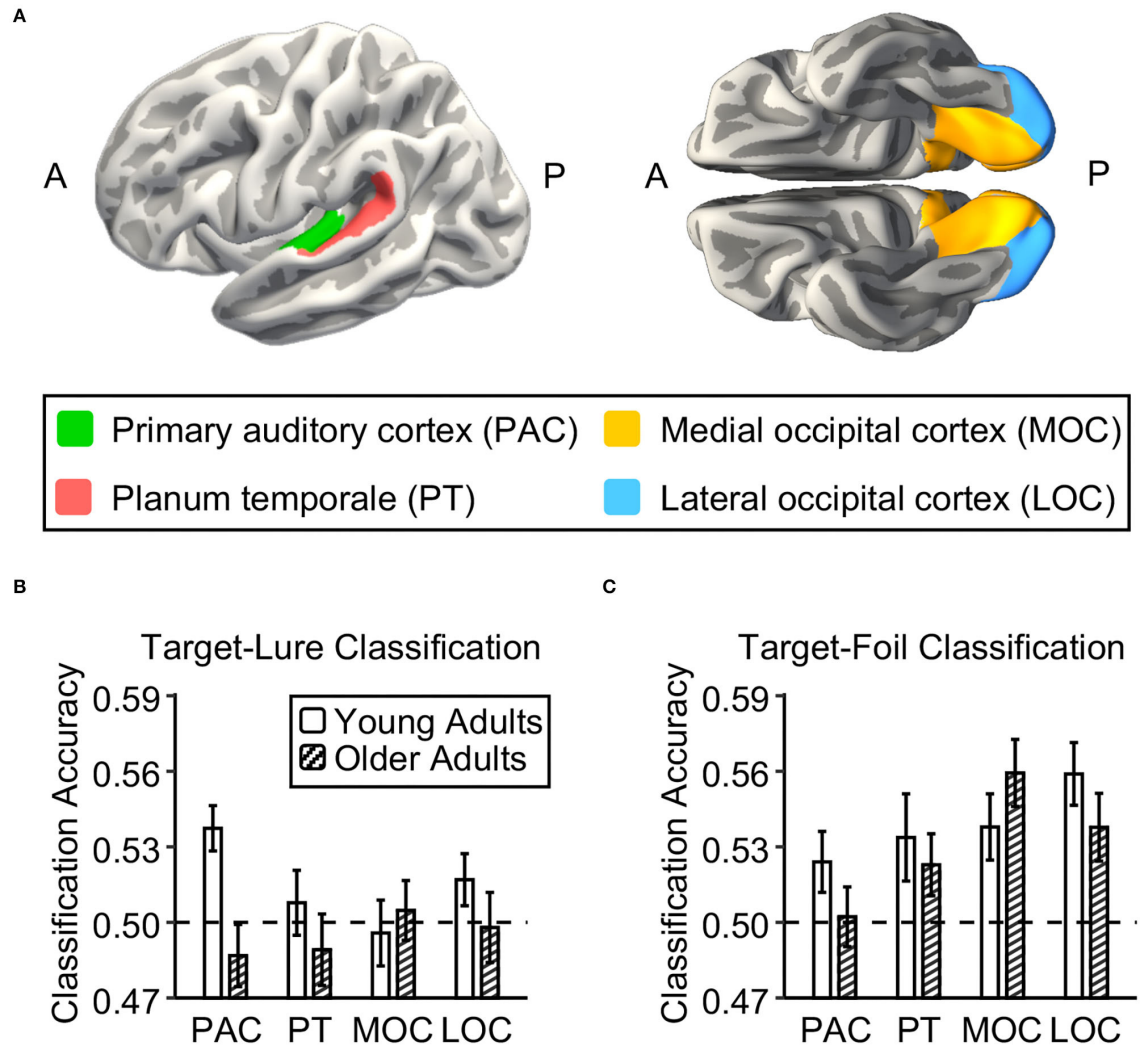
Neural discriminability in auditory and visual cortex in young and older adults. **(A)** Four brain regions of interest (ROIs) in the auditory and visual cortex were defined using Freesurfer segmentation. Two auditory ROIs were the primary auditory cortex (PAC; green) and planum temporale (PT; red). Two visual ROIs were the medial occipital cortex (MOC; yellow) and the lateral occipital cortex (LOC; blue). **(B,C)** Bar graphs show the mean accuracy for the classifier trained to distinguish between targets and lures and to distinguish between targets and foils in each ROI in young adults and in older adults, respectively. The dashed lines indicate the theoretical chance level. The error bars indicate the standard errors of the means.

To obtain anatomical ROIs in the native space, we segmented and parceled the structural image for each participant using FreeSurfer (version 6.0) (Figure 2A and Supplementary Figure 2). Following the previous study of Du et al. (2016), based on the Destrieux atlas, we defined two ROIs within the auditory cortex, including the primary auditory cortex (PAC; a combination of Heschl's gyrus and transverse temporal sulcus) and planum temporale (PT). Following the previous study of Bowman et al. (2019), based on the Desikan-Killiany atlas, we defined two ROIs within the visual cortex, including the medial occipital cortex (MOC, a combination of the lingual gyrus and cuneus) and lateral occipital cortex (LOC). All four ROIs were collapsed across both hemispheres.

To examine whether the neural patterns of targets in a given ROI are distinguishable from lures and/or from foils, the one-tailed one-sample *t-*test was used to examine whether its classification accuracy was significantly higher than the chance level (> 50%) in young and older adults, separately. Next, to examine the age effect on the classification accuracy in each ROI, we conducted a linear regression analysis using the age group (older adults = −1 and young adults = 1) as an independent variable and the neural classification accuracy (i.e., either for target-lure or for target-foil) as a dependent variable. Additionally, we added the univariate activation differences of conditions (i.e., targets minus lures or targets minus foils) for each participant in each ROI as a nuisance covariate in the regression model to confirm if the age difference in neural classification accuracy was not due to the age difference in the univariate activation level. For example, we added the activation difference of targets and lures for each participant as a nuisance covariate to the regression model with target-lure classification being the dependent variable.

Furthermore, we examined whether neural discriminations for each ROI were related to behavioral performance (i.e., true and false recognition) using regression analysis. In the regression model, we used the age group (older adults = −1 and young adults = 1), neural target-lure and target-foil classification accuracy in the ROI, age group × target-lure interaction term, and age group × target-foil interaction term as independent variables and the behavioral performance as a dependent variable, adding the univariate activation differences (i.e., targets minus lures and targets minus foils) in the ROI as nuisance covariates. For regression analyses, we corrected the *p*-value for the overall regression model of four ROIs. When there is a significant interaction effect, we used correlation analysis to clarify how age moderated the relationship between behavioral performance and neural discriminations. It should be noted that based on the sample size of 27 for each group, the detectable smallest correlation is 0.51 (alpha = 0.05, power = 80%).

Finally, the whole-brain searchlight analysis from the brainiak package (v0.10) in Python was used to explore whether there would be any other brain regions showing age differences in the neural discriminability between targets and lures and between targets and foils. The voxel-based searchlight analysis followed the same classification procedure as above. The 5 × 5 × 5 cubic searchlight method was used. For each center voxel, we extracted neural signals from the cubic ROI including 125 surrounding voxels for classification, and then, we generated whole-brain accuracy maps for target-lure and target-foil classifications in the standard space for each participant. Using FSL's Randomize function, we conducted group-level statistics of searchlight analyses (Winkler et al., 2014). To identify brain regions showing both above-chance classification and their age differences, the 10,000 iterations of the *t-*test were used to generate four contrasts (i.e., young adults > older adults, older adults > young adults, young adults > chance, and older adults > chance). We used the threshold-free cluster enhancement within a gray matter mask, which was defined from the Harvard-Oxford atlas (thresholded at a probability of 25%), with a variance smoothing of 2 mm. To ensure these brain regions showing age differences also have the above-chance classification accuracy in at least one of two age groups, we reported the results of age group differences, masking with regions based on the contrast of appropriate age group > chance. For example, the young adults > older adults contrast was inclusively masked with the young adults > chance contrast. Additionally, we used the threshold of *t* > 1.7 (*p* < 0.05) and a minimum cluster extent of 10 voxels.

### Univariate Activation-Based Analysis

Based on our previous studies on young adults (Ye et al., 2016; Zhu et al., 2019), we focused on the monitoring process in the left lateral prefrontal cortex during retrieval. Using the General Linear Model (GLM), two types of trials (i.e., Lure and Foil) were modeled. Target trials, the reaction time, six motion parameters, and frame-wise displacement were treated as nuisance variables. The contrast of Lure–Foil was defined to examine the effect of the monitoring process for each run. The fixed-effects model was used to calculate the cross-run contrast of Lure–Foil for each participant in a higher-level analysis. This contrast was then fed to group analysis with a random-effects model using full FMRIB's Local Analysis of Mixed Effect 1+2 with automatic outlier detection. Group images were thresholded using cluster detection analysis (with a threshold of Z > 2.3 and a cluster probability of *p* < 0.05). Since we only focused on the monitoring process in the left lateral prefrontal cortex, the thresholded group images were corrected for multiple comparisons in a mask of the left lateral prefrontal cortex using small-volume correction. To explore the monitoring process in each age group, we compared the univariate activation of lures and foils in young and older adults, separately. Then, we compared activation differences between young and older adults in the monitoring process [i.e., YA (Lure–Foil) – OA (Lure–Foil)]. The brain region showing age differences in the monitoring process was defined as the ROI, by including all of the voxels in this cluster showing suprathreshold activation for this contrast. The univariate activation of this ROI for lures and foils was then extracted and further analyzed. Independent and paired *t*-tests were used to examine the nature of the interaction between age group and item type. Finally, we explored the correlation between the univariate activation for lures in the ROI and behavioral performance of false recognition (i.e., the proportion of “old” responses to lures and the propensity for high-confidence false recognition).

Moreover, we conducted regression analyses to explore the relationship between neural discriminations in the sensory cortex (target-lure classification) and the monitoring process in the left prefrontal cortex (activation for lures). For each ROI, we used the age group (older adults = −1 and young adults = 1), neural target-lure classification accuracy, and their interaction term as the independent variables, the activation level for the lure in the left prefrontal cortex as a dependent variable, adding the univariate activation differences (i.e., targets minus lures) in the ROI as a nuisance covariate. We did not explore the relationship between the LPFC monitoring process for lures and neural classification of target-foil because the LPFC monitoring process for lures should not impact discriminating the neural patterns between targets and foils.

## Results

### Behavioral Results

In both young and older adults, true recognition was higher than false recognition, which in turn was higher than foil recognition (i.e., the proportion of “old” responses: target > lure > foil, *ps* < 0.001) (Figure 1B and Supplementary Table 1). Critically, older adults have lower true recognition than young adults, *t*_(52)_ = −5.62, *p* < 0.001. But there was no age group difference in false recognition, *t*_(52)_ = −0.77, *p* = 0.44, and foil recognition, *t*_(52)_ = 0.77, *p* = 0.44. In older adults, there was a positive correlation between true and false recognition, *r*_(25)_ = 0.61, *p* < 0.001, but no correlation was found between true and foil recognition, *r*_(25)_ = 0.07, *p* = 0.72. In young adults, true recognition did not correlate with false recognition, *r*_(25)_ = 0.13, *p* = 0.52, or with foil recognition, *r*_(25)_ = −0.16, *p* = 0.42. Regarding the reaction time at retrieval, older adults were slower to recognize targets and reject foils than young adults (*ps* < 0.01), but there was no age difference in reaction time for false recognition (*p* = 0.12) (Supplementary Table 1).

Regarding the propensity for high-confidence recognition, young adults made more high-confidence “old” responses to targets than older adults, whereas older adults made more high-confidence “old” responses to lures and foils than young adults. Compared to young adults, older adults have a lower propensity for high-confidence true recognition, *t*_(52)_ = −4.88, *p* < 0.001, but a higher propensity for high-confidence false and foil recognition, *t*_(52)_ = 4.31 and 3.07, *ps* < 0.004 (Figure 1C and Supplementary Table 2). These results suggest that older adults have reduced true memory and stable false memory, but display overconfidence for false alarms relative to young adults.

### Age Deficits in Neural Discriminability in the Primary Auditory Cortex

To examine age differences in neural discriminability in the sensory cortex, we tested whether the neural activation patterns in four ROIs of the visual and auditory cortex could be used to distinguish targets from lures and foils in each age group and whether they differed with age. Regarding the classification accuracy for targets vs. lures (Figure 2B), we first compared the target-lure classification accuracy to theoretical chance (0.5) in each ROI in each age group. In young adults, the neural activation pattern in the primary auditory cortex can be used to distinguish between targets and lures at above-chance levels [*t*_(26)_ = 3.77, *p* = 0.0004], but not in the other three ROIs [*t*_(26)_ = 0.16, 0.47, and 1.49, *ps* = 0.44, 0.32, and 0.07 for planum temporale, medial occipital cortex, and lateral occipital cortex, respectively]. However, in older adults, none of these four ROIs showed above-chance performance for the classification between targets and lures [*t*_(26)_ = −0.79, −0.36, −0.48, and −0.04, *ps* = 0.78, 0.64, 0.68, and 0.51 for primary auditory cortex, planum temporale, medial occipital cortex, and lateral occipital cortex, respectively]. There was a significant age difference in the target-lure classification accuracy for the primary auditory cortex [*t*_(52)_ = 2.86, *p* = 0.006], but not for the other three ROIs [*t*_(52)_ = 0.38, 0.67, and 0.92, *ps* = 0.71, 0.51, and 0.36, for planum temporale, medial occipital cortex, and lateral occipital cortex, respectively]. After controlling for the univariate activation in each ROI, there was a significant age difference in the target-lure classification accuracy for the primary auditory cortex [*t*_(52)_ = 2.72, *p* = 0.009], but not for the other three ROIs [*t*_(52)_ = 0.37, 0.78, and 0.96, *ps* = 0.72, 0.44, and 0.34, for planum temporale, medial occipital cortex, and lateral occipital cortex, respectively]. After correcting for multiple comparisons for four ROIs, the target-lure classification accuracy in the primary auditory cortex in young adults was still at above-chance levels (FDR corrected *p* = 0.003), and it was still higher than that in older adults after controlling for univariate activation (FDR corrected *p* = 0.04).

Regarding the classification accuracy for targets vs. foils (Figure 2C), we first compared the target-foil classification accuracy to theoretical chance (0.5) in each ROI in each age group. In young adults, the neural activation pattern in each ROI can be used to distinguish between targets and foils at above-chance levels [*t*_(26)_ = 1.87, 2.23, 3.13, and 4.58, *ps* = 0.04, 0.02, 0.002, and 0.0001, for primary auditory cortex planum temporale, medial occipital cortex, and lateral occipital cortex, respectively]. In older adults, the neural activation pattern in the visual cortex can be used to distinguish between targets and foils at above-chance levels [*t*_(26)_ = 4.21 and 2.94, *ps* = 0.0001 and 0.003, for medial and lateral occipital cortexes, respectively], but not for the auditory cortex [*t*_(26)_ = 0.30 and 1.46, *ps* = 0.38 and 0.08 for primary auditory cortex and planum temporale, respectively]. After correcting for multiple comparisons for four ROIs, the target-foil classification accuracy in each of four ROIs in young adults was still at above-chance levels (FDR corrected *ps* < 0.04), while the target-foil classification accuracy in the visual cortex in older adults was still at above-chance levels (FDR corrected *ps* < 0.006). There were no age differences in target-foil classification accuracy in the four ROIs [*t*_(52)_ = 1.11, 0.88, −0.88, and 0.96, *ps* = 0.27, 0.38, 0.38, and 0.34, for primary auditory cortex, planum temporale, medial occipital cortex, and lateral occipital cortex, respectively]. After controlling for the univariate activation in each ROI, none of these four ROIs show age difference in the target-foil classification accuracy [*t*_(52)_ = 1.17, 0.50, −0.96, and 1.10, *ps* = 0.25, 0.62, 0.34, and 0.27, for primary auditory cortex, planum temporale, medial occipital cortex, and lateral occipital cortex, respectively].

We further explored whether age group moderated the relationship between behavioral performance (i.e., true and false recognition) and neural classification accuracy (i.e., target-lure and target-foil classification) in each ROI (Supplementary Table 3). In primary auditory cortex, the overall model for true recognition reached significance, *F*_(5, 48)_ = 9.35, FDR corrected *p* < 0.001, *R*^2^ = 0.49, with the age group × target-lure classification term reaching significance (*p* = 0.02). Further correlation analyses between true recognition and target-lure classification revealed that there was a significant positive correlation in young adults, *r*_(25)_ = 0.39, *p* = 0.046, whereas there was a marginally significant negative correlation in older adults, *r*_(25)_ = −0.35, *p* = 0.075. Although the overall models were also significant in planum temporale [*F*_(5, 48)_ = 8.29, FDR corrected *p* < 0.001, *R*^2^ = 0.46], medial occipital cortex [*F*_(5, 48)_ = 6.68, FDR corrected *p* < 0.001, *R*^2^ = 0.41], and lateral occipital cortex [*F*_(5, 48)_ = 9.00, FDR corrected *p* < 0.001, *R*^2^ = 0.48], they did not reveal any significant interaction terms. The overall models for false recognition did not reach significance in four ROIs (FDR corrected *ps* > 0.24). After controlling for activation level, the results were similar to those before controlling. These results suggested that neural discriminability between targets and lures in the primary auditory cortex declined with age, and that it was positively correlated with true recognition in young adults but not in older adults.

### Whole-Brain Searchlight Analysis of Age Deficits in Neural Discriminability

**Figure 3:**
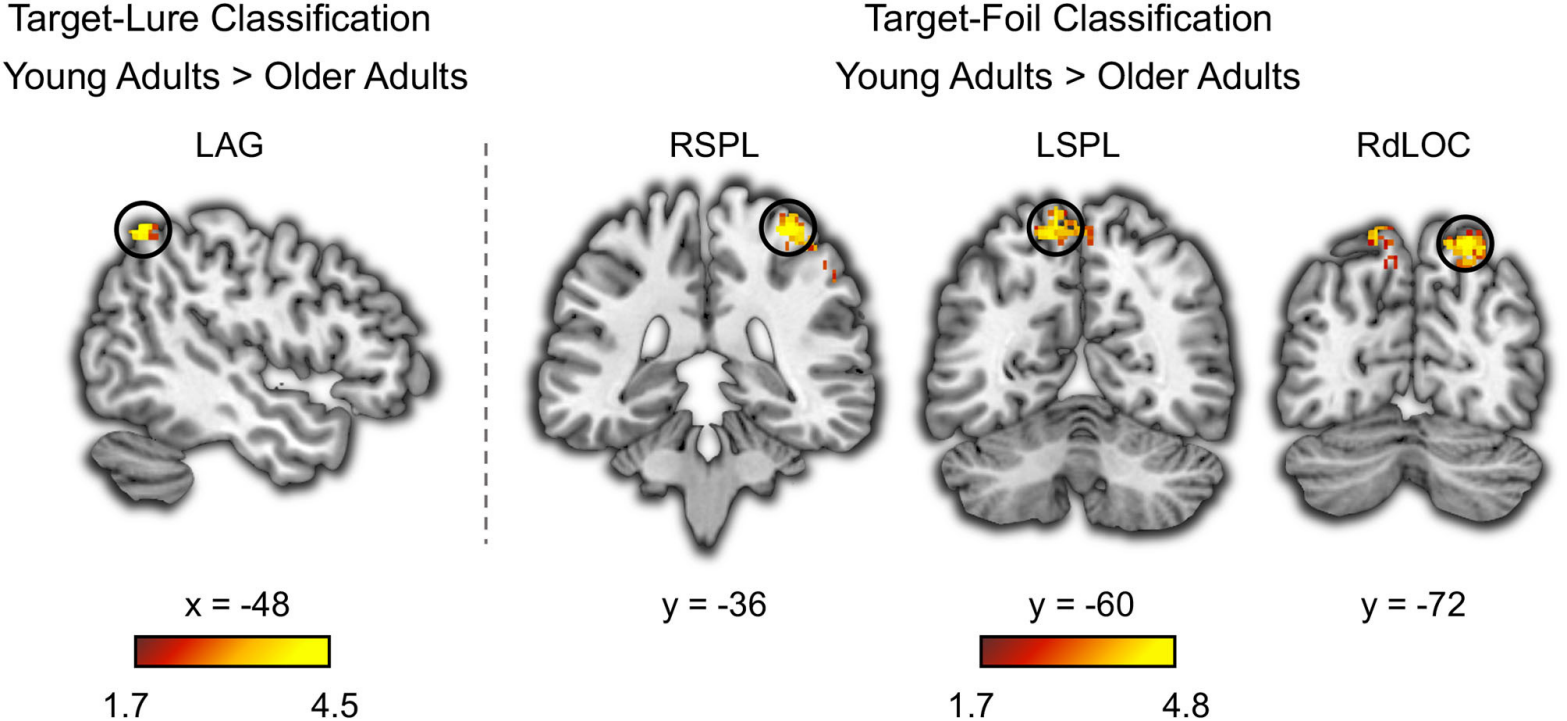
Age deficits in neural discriminability based on whole-brain searchlight classification analysis. The left angular gyrus (LAG) showed age deficits in the neural pattern classifications accuracy for distinguishing between targets and lures. The right superior parietal lobe (RSPL), the left superior parietal lobe (LSPL), and the right dorsolateral occipital cortex (RdLOC) showed age deficits in the neural pattern classifications accuracy for distinguishing between targets and foils. Neural discriminability in these brain regions was at above-chance level in young adults, and it was higher in young adults compared to older adults.

Based on the whole-brain searchlight analysis, we found that several brain regions outside the sensory cortex showed above chance classification of target-lure in young adults and target-foil in both age groups (Supplementary Figure 3 and Supplementary Table 4). Several other brain regions also showed age deficits in target-lure and target-foil classifications (Figure 3). The target-lure classification accuracy in the left angular gyrus (MNI: x = −48, y = −58, z = 58, k = 86, *t* = 4.46) was higher in young adults compared with older adults. Moreover, the target-foil classification accuracy in the occipito-parietal cortex, including the right superior parietal lobe (MNI: x = 36, y = −36, z = 66, k = 840, *t* = 4.80), the left superior parietal lobe (MNI: x = −10, y = −60, z = 66, k = 398, *t* = 4.32), and the right dorsolateral occipital cortex (MNI: x = 22, y = −72, z = 58, k = 127, *t* = 4.73), was higher in young adults compared with older adults. No brain regions showed higher target-lure or target-foil classification accuracy in older adults compared with young adults.

### Age Deficits in Prefrontal Monitoring for Lures

**Figure 4:**
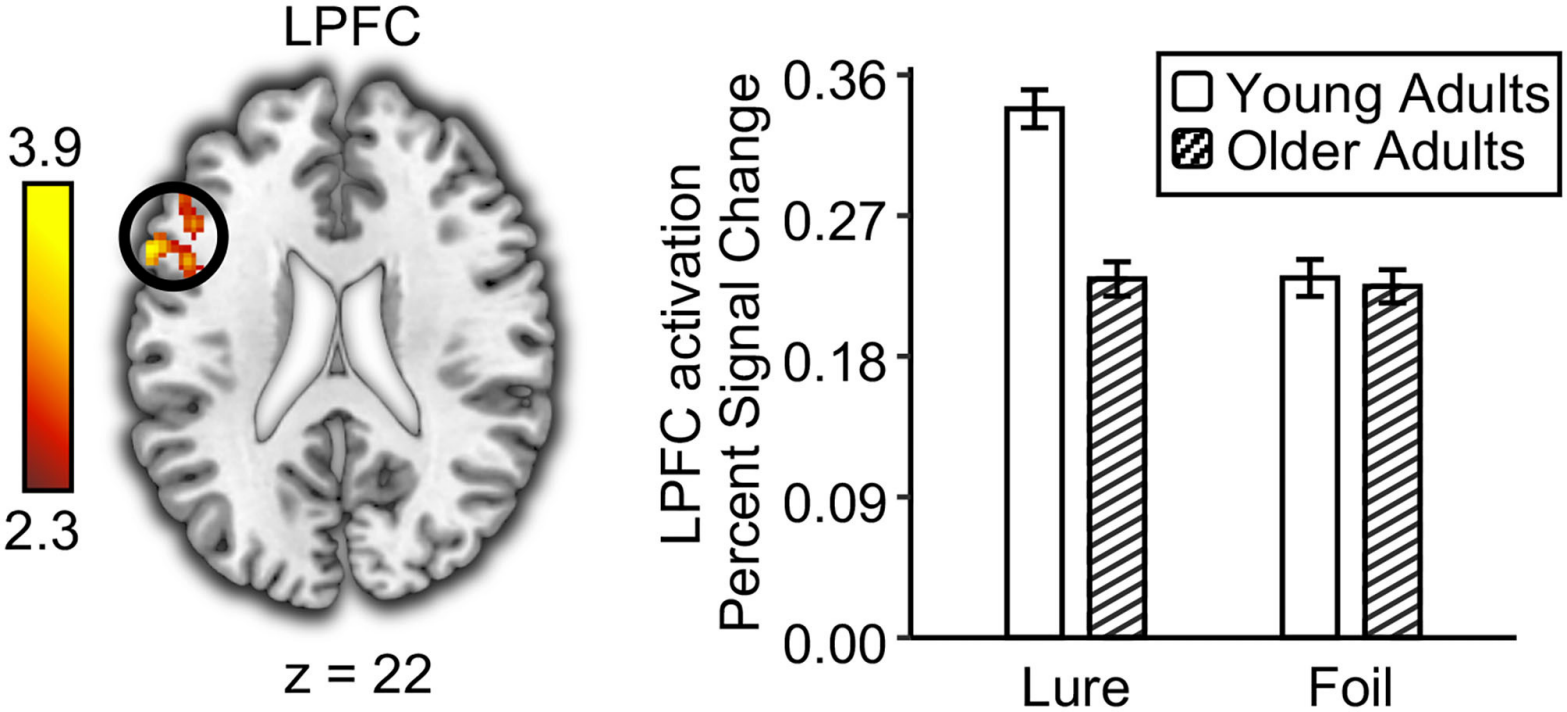
Age deficits in prefrontal monitoring process at retrieval. The left lateral prefrontal cortex (LPFC) showed greater univariate activation to lures than to foils in young adults compared to older adults. The bar graph shows the univariate activation level in the left lateral prefrontal cortex for lures and foils in young and older adults. Error bar indicates standard errors of the means.

To examine age differences in the prefrontal monitoring process, we tested whether the neural activation for lures was higher than that for foils (i.e., activation: lure > foil) in each age group and whether they differed with age. Although both young and older adults showed greater activations to lures compared with foils (Supplementary Figure 4), direct comparison between young and older adults in this contrast revealed greater activation differences in the left lateral prefrontal cortex (LPFC; MNI: −60, 16, 22, Z = 3.9) (Figure 4). However, no brain region showed a greater monitoring process in older adults compared to young adults. Specifically, the LPFC activation for lures was higher than that for foils in young adults, *t*_(26)_ = 6.42, *p* < 0.001, but not in older adults, *t*_(26)_ = 0.33, *p* = 0.75. Independent *t*-test showed an age deficit in the LPFC activation for lures, *t*_(52)_ = 2.44, *p* = 0.02, but not for foils, *t*_(52)_ = 0.14, *p* = 0.89. Next, we explored whether the prefrontal monitoring for lures was related to behavioral performance for lures (i.e., false recognition and the propensity of high-confidence false recognition) in each age group, separately. Results showed that the LPFC activation for lures had a negative correlation with the propensity for high-confidence false recognition in young adults, *r*_(25)_ = −0.38, *p* = 0.048, but not in older adults, *r*_(25)_ = 0.05, *p* = 0.79. However, the LPFC activation for lures did not correlate with false recognition in young and older adults [*r*_(25)_ = −0.21 and 0.16, *ps* = 0.28 and 0.42 for young and older adults, respectively]. Together, these results suggest that prefrontal monitoring for lures declined with age, which helped to suppress the propensity for high-confidence false recognition in young adults but not in older adults.

Finally, we explored whether age moderates the relationship between prefrontal monitoring for lures and neural classification accuracy of target-lure for each ROI (Supplementary Table 5). In four ROIs, no significant overall model was found (FDR corrected *ps* > 0.06).

## Discussion

Extending previous studies showing age-related neural dedifferentiation for visual memory (Bowman et al., 2019; Koen et al., 2020; Sommer and Sander, 2022), our findings suggest that age differences in true and false memories following auditory learning are associated with reduced neural discriminability in primary auditory cortex and reduced prefrontal monitoring during memory retrieval. The present study used multivariate and univariate analyses to investigate the aging effect on neural discriminability in the sensory cortex and prefrontal monitoring process in a visual recognition test after hearing words. Behavioral results revealed that older adults showed decreased true recognition but increased propensity for high-confidence false recognition compared with young adults. Regarding neural discrimination, only the primary auditory cortex showed age deficits in neural discriminability between targets and lures during memory retrieval after listening to words. Regarding the monitoring process, the left lateral prefrontal cortex showed age deficits in the monitoring process for lures. As indicated by the neural-behavioral correlations in young adults, more distinctive representations in the primary auditory cortex during memory retrieval were associated with better true recognition, whereas stronger prefrontal monitoring for lures was associated with reduced propensity for high-confidence false recognition.

### Age Effects on Neural Discriminability and Their Relationship to True Memory

Using the DRM paradigm with auditory encoding and visual testing, we revealed that neural activation patterns in the primary auditory cortex could be used to distinguish targets and lures during memory retrieval in young adults but not in older adults. Meanwhile, there was no age difference in neural discriminability between targets and foils in the auditory and visual cortex. Targets and foils differ in both sensory and semantic details, whereas targets and lures differ only in sensory details. Supporting the sensory reactivation hypothesis (Slotnick and Schacter, 2004), true memory of auditory information was accompanied by greater retrieval of auditory details in the primary auditory cortex than false and foil memory. Although prior studies in young adults have shown greater activations to targets than lures in large portions of the auditory cortex and nearby regions (Schacter et al., 1996; Abe et al., 2008), we found that neural patterns that distinguish targets from lures and foils during memory retrieval selectively occurred in the primary auditory cortex. Specifically, Heschl's gyrus and transverse temporal sulcus rather than planum temporale are implicated in the retention of highly detailed auditory representations (Du et al., 2016), which facilitate distinguishing targets from lures and foils in young adults.

Compared to young adults, older adults have lower true recognition and more difficulty in distinguishing targets from lures after listening to words, as shown in the current and previous behavioral studies (Kensinger and Schacter, 1999; Smith et al., 2005). Using the decoding method, we found that neural patterns in the auditory cortex cannot distinguish targets from lures and foils for older adults. It suggested that age effect on behavioral performance is associated with deficits in the neural discriminability of true and false memories in the primary auditory cortex during memory retrieval. Previous studies of auditory perception have shown age-related neural dedifferentiation in large portions of the auditory cortex (Du et al., 2016; Lalwani et al., 2019; Erb et al., 2020). However, our study suggested that only the primary auditory cortex showed age-related neural dedifferentiation during memory retrieval. Together with prior work on visual memory (Bowman et al., 2019), our findings indicate that age deficits in neural discriminability between highly similar items during memory retrieval are confined to the primary sensory cortex involved in encoding.

In addition, we found that the relationship between true recognition and neural discrimination in the primary auditory cortex was moderated by age, which is different from prior work on visual memory showing age-invariant neural-behavioral correlations (Bowman et al., 2019; Koen et al., 2019). To our knowledge, no studies have examined the relationship between neural discriminability and auditory memory performance. The correlation between age-related neural dedifferentiation and memory performance may be different depending on the sensory modality of the studied items. In our study, young adults showed a positive correlation between true recognition and target-lure classification accuracy in the primary auditory cortex, whereas older adults showed a negative correlation at a trend level. It suggests that the primary auditory cortex may carry different auditory information and is involved in different cognitive processes during memory retrieval in each age group. In young adults, the positive correlation can be interpreted as evidence that the primary auditory cortex carries distinctive auditory representations of studied words, which facilitates true recognition during memory retrieval. In older adults, the negative correlation at a trend level may reflect the auditory hallucination of unstudied lures evoked by the primary auditory cortex. Older adults with higher true recognition may be more likely to claim to remember how lures sounded in the study, which resulted in reduced neural discriminability between targets and lures in this brain region.

As for the visual cortex, we found that the neural patterns of targets and lures in the medial and lateral visual cortexes were not distinguishable in both age groups, which is not consistent with our hypothesis. It suggested that reactivation of visual mental imagery did not contain sensory details to distinguish targets from lures. It has been shown that the primary visual cortex contains high-resolution mental imagery representations when the task requires an object or spatial processes which involved more low-level visual features (e.g., lines, angle, color, etc.) (Kosslyn et al., 1995; Thompson and Kosslyn, 2000; Kosslyn and Thompson, 2003). Thus, the loss of detail in visual mental imagery may be due to the materials we used in the current study. Another possible reason is that perceptual judgment tasks may affect the activation pattern of the target in the visual cortex. Although it has been shown that perceptual judgment tasks do not affect the activity level of memory task signals (Stark and Squire, 2001), it remains an open question whether there is an effect on pattern classification. Future research could investigate this possibility using an experimental design with other baseline tasks. Besides, we found the medial and lateral visual cortexes showed above-chance classification of target-foil in both age groups but did not show age differences. It indicated that reactivation of mental imagery of listened words in the visual cortex contains general sensory information but without details. Consistent with the previous study (Bowman et al., 2019), the ability to represent general sensory information that lacks sufficient details did not decline with age.

Besides the sensory cortex, the whole brain searchlight analysis showed age deficits in target-lure classification in the left angular gyrus and age deficits in target-foil classification in the bilateral superior parietal lobe. It indicated differential involvement of the angular gyrus and the superior parietal lobe in recollection and familiarity (Wagner et al., 2005; Cabeza et al., 2008). Specifically, the left angular gyrus was involved in the recollection of episodic details and multisensory integrations (Kuhl and Chun, 2014; Thakral et al., 2017), whereas the superior parietal lobe was involved in familiarity-based recognition (i.e., a sense that the word has been studied but without details) (Vilberg and Rugg, 2008; Zhu et al., 2019). As further evidence of functional differences between these two brain regions, repetitive transcranial magnetic stimulation over the left angular gyrus, but not the superior parietal lobe, modulates speech comprehension in challenging auditory conditions (Hartwigsen et al., 2015). Moreover, age deficits in the parietal cortex may contribute to memory loss in older adults. As shown in a recent study, the left angular gyrus was involved in the recollection effect in young adults but not in older adults (Hou et al., 2022). Consistent with these previous studies, we found that the neural activation pattern in the left angular gyrus could be used to distinguish targets and lures during memory retrieval in young adults but not in older adults.

### Age Effects on Prefrontal Monitoring and Their Relationship to the Propensity for High-Confidence False Memory

Compared with young adults, older adults showed impaired prefrontal monitoring process for unheard lures during retrieval, which contributed to age-related increases in the propensity for high-confidence false recognition. On the behavioral level, we found that older adults have an increased propensity for high-confidence false recognition after listening to words than young adults. Replicating previous aging studies using visual stimuli to examine false memory (Dennis et al., 2007, 2008; Sikora-Wachowicz et al., 2021), older adults had a greater propensity for high-confidence false recognition compared to young adults, but there was no age difference in false recognition. The age-related increase in the propensity for high-confidence false recognition may reflect age deficits in strategic retrieval processes (Shing et al., 2009; Fandakova et al., 2013).

On the neural level, we found that older adults showed lower activations in the left lateral prefrontal cortex to lures than young adults. This age difference in prefrontal monitoring processes is consistent with results from previous aging studies using visual stimuli (Dennis et al., 2007, 2008; Kurkela and Dennis, 2016; Sikora-Wachowicz et al., 2021). As shown in our previous studies using the same experimental design (Ye et al., 2016; Zhu et al., 2019), the left lateral prefrontal cortex exhibited greater activation to lures than to foils judged as new during memory retrieval in young adults. For young adults, our previous study revealed that prefrontal monitoring for lures during memory retrieval was weaker after auditory learning than after visual learning, resulting in increased false recognition (Zhu et al., 2019). This may be due to the fact that the prefrontal monitoring process is triggered by the discrepancy between semantic and sensory memory signals. Compared to the visual learning and visual test condition, prefrontal monitoring for lures in the visual test following the auditory learning was diminished because the discrepancy between semantic and sensory signals was smaller. Extending previous studies, the current findings indicated that prefrontal monitoring for lures was further reduced in older adults compared to young adults during the visual recognition test following auditory learning. Compared to young adults, older adults may find it even harder to detect the differences between semantic and sensory signals. Moreover, aging may impair the ability to monitor the source of information during retrieval (McDonough and Gallo, 2013; Devitt and Schacter, 2016). Overall, age differences in prefrontal activation to lures reflect the impairment of monitoring processes during memory retrieval in older adults (Devitt and Schacter, 2016; Fandakova et al., 2018).

Regarding the neural-behavioral correlation, we found a negative correlation between prefrontal monitoring for lures and the propensity for high-confidence false recognition in young adults but not in older adults. Specifically, lower activations for lures in the left lateral prefrontal cortex were associated with a higher propensity for high-confidence false recognition in young adults. It should be noted that this neural-behavioral correlation was found for the propensity for high-confidence false recognition rather than for the endorsement rate of false recognition. These results further suggest that this brain region is involved in subjective memory decisions that reduce high-confidence memory errors, rather than in semantic processing that leads to false recognition (Chua et al., 2004). Previous behavioral studies have shown that individuals with higher executive function had lower false memory with high confidence for both young and older adults (Butler et al., 2004; Peters et al., 2006; Chan and McDermott, 2007; Fandakova et al., 2013). However, we did not find the neural-behavioral correlation in older adults, probably due to the limited sample size. Future research could consider using larger sample sizes to further explore the relationship between prefrontal activation and high-confidence false memories in older adults.

## Conclusion

In sum, our findings indicated that age differences in true and false memories following auditory learning were associated with reduced neural discriminability in the primary auditory cortex and reduced prefrontal monitoring at retrieval. Individual differences analysis further showed that more distinctive representation in the primary auditory cortex during memory retrieval was associated with better true recognition, whereas stronger prefrontal monitoring was associated with reduced propensity for high-confidence false recognition in young adults only. Further exploration of these two neural mechanisms we have identified should help us to better understand the aging effect on auditory memory.

## Data Availability Statement

The raw data supporting the conclusions of this article will be made available by the authors, without undue reservation.

## Ethics Statement

The studies involving human participants were reviewed and approved by Institutional Review Board of the State Key Laboratory of Cognitive Neuroscience and Learning at Beijing Normal University, China. The patients/participants provided their written informed consent to participate in this study.

## Author Contributions

BZ, XS, and WL designed research. XS, WL, and YG collected data. BZ, XS, and YG analyzed data. BZ and XS wrote the article. All authors contributed to the article and approved the submitted version.

## Funding

This study was supported by the National Natural Science Foundation of China (31730038, 31971000, and 31571132).

## Conflict of Interest

The authors declare that the research was conducted in the absence of any commercial or financial relationships that could be construed as a potential conflict of interest.

## Publisher's Note

All claims expressed in this article are solely those of the authors and do not necessarily represent those of their affiliated organizations, or those of the publisher, the editors and the reviewers. Any product that may be evaluated in this article, or claim that may be made by its manufacturer, is not guaranteed or endorsed by the publisher.
