# Supplementary material for: Age Effects on Neural Discriminability and Monitoring Process During Memory Retrieval for Auditory Words
Source: Front Aging Neurosci. 2022 Jul 19;14:884993. doi: 10.3389/fnagi.2022.884993 (PMC9343999; doi:10.3389/fnagi.2022.884993)
Supplement: Supplementary file 1 [file Data_Sheet_1.docx]

Supplementary Material

# Supplementary Figures and Tables

**Number of figures**: 4

**Number of tables**: 5

## Supplementary Figures

**Supplementary Figure 1**. Neural activation comparisons between memory task and perceptual judgment (arrows orientation) task at retrieval. **(A)** To test whether the perceptual judgment task is an appropriate baseline for memory task as reported by Stark and Squire (2001), we conducted univariate analyses for the contrast of memory task and perceptual judgment task in the whole brain for both age groups. The results revealed higher activation of memory task than perceptual judgment task in distributed regions, including the sensory cortex, frontal lobe, and parietal lobe. Besides, we found greater negative activation for memory task than perceptual task in the inferior parietal gyrus and medial prefrontal cortex (i.e., retrieval: memory task < perceptual task) and their neural activation levels had negative values for both perceptual judgment and memory tasks at retrieval (i.e., retrieval: memory task < perceptual task < 0). This may be due to the fact that these two regions are parts of the default mode network. In our single-trial estimation model, the perceptual judgment was not included in the model but was treated as an implicit baseline. Consistent with the previous study (Stark and Squire, 2001), these results suggested that using the perceptual judgment task is an appropriate baseline for memory task. **(B)** To test whether there was a difference in sensitivity (i.e., memory task minus perceptual judgment task) in sensory cortex, we conducted a mixed repeated ANOVA with the factor of groups (young adults and older adults) by ROIs (primary auditory cortex [PAC] and planum temporale [PT], medial occipital cortex [MOC] and lateral occipital cortex [LOC]). Results showed that there were significant main effects of age group and ROIs (*F*(1, 52) = 16.24 and *F*(3, 156) = 76.65, *ps* < 0.001), but the interaction was not significant (*F*(3, 156) = 3.04, *p* = 0.06). The post hoc *t*-test showed that the sensitivity to memory task in the visual cortex (i.e., MOC and LOC) was higher than that in the auditory cortex (i.e., PAC and PT) (*ps* < 0.02). They suggested that there was a higher sensitivity to memory task in the visual cortex compared to the auditory cortex on the activation level.


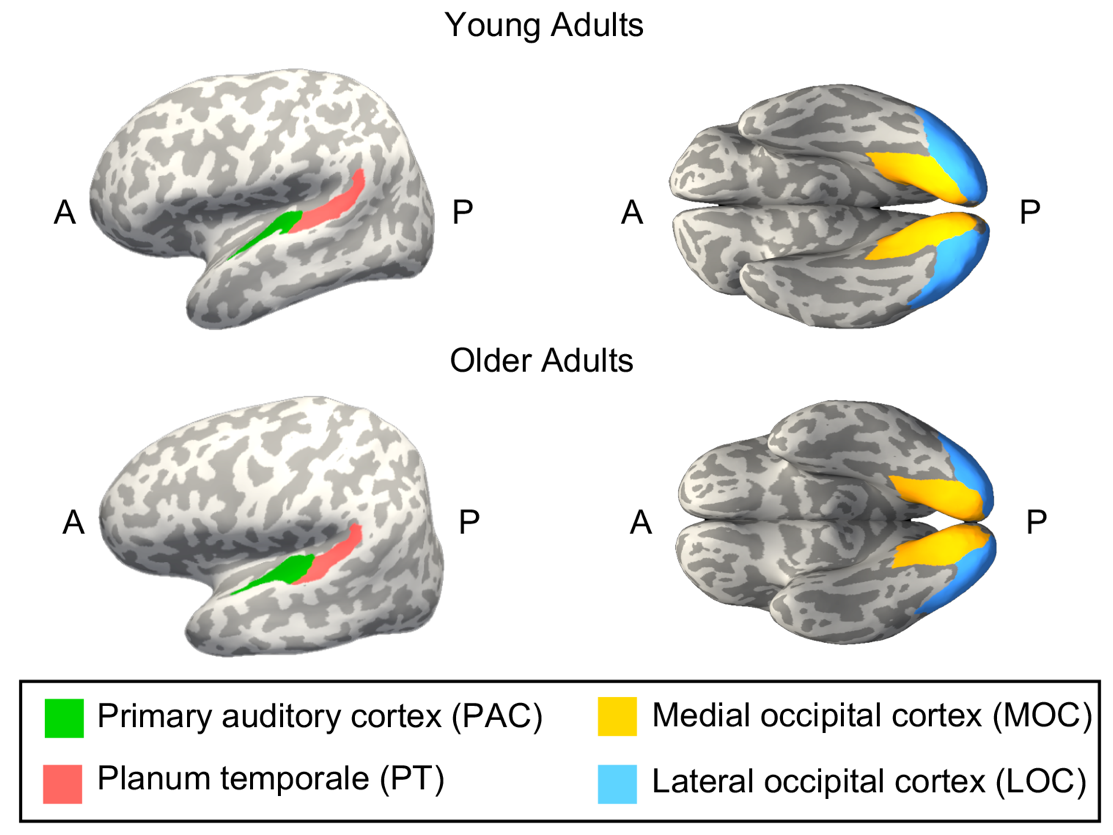


**Supplementary Figure 2.** Brain regions of interests (ROIs) were displayed for a representative young adult (top panel) and a representative older adult (bottom panel). Two auditory ROIs were the primary auditory cortex (PAC; green) and planum temporale (PT; red). Two visual ROIs were the medial occipital cortex (MOC; yellow) and lateral occipital cortex (LOC; blue).

**Supplementary Figure 3.** The results of whole-brain searchlight analysis of target-lure classification (target-lure > 50%) and target-foil classification (target-foil > 50%) for each age groups. No brain regions showed above chance for target-lure in older adults. It reflected the dedifferentiation of targets and lures for older adults and mirrored the behavioral result that true memory was correlated with false memory (*r*(25) = 0.61, *p* < 0.001).

**
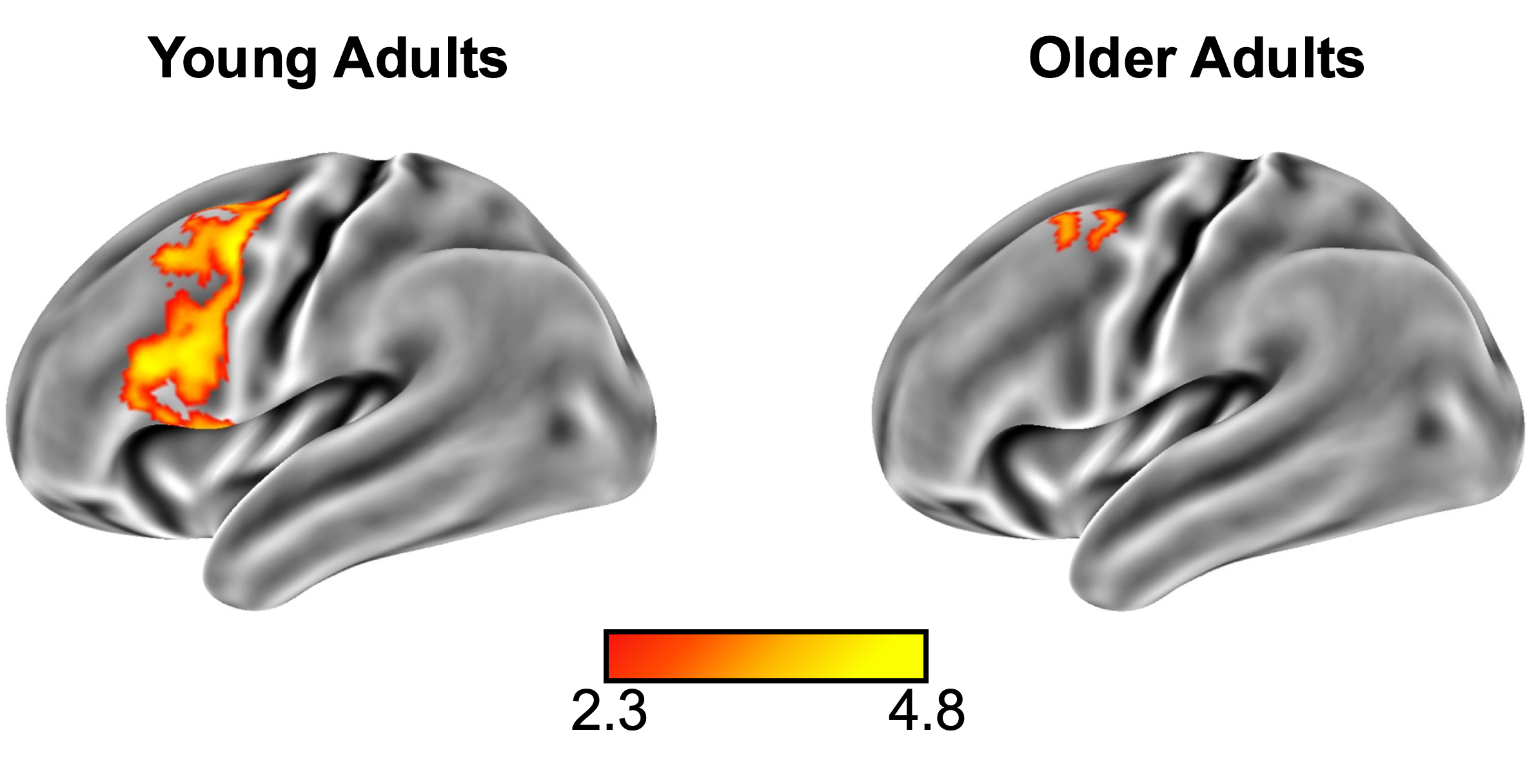
**

**Supplementary Figure 4.** The left lateral prefrontal cortex showed greater activation to lures as compared with foils in young adults (MNI: -60, 18, 24, Z = 4.8) and older adults (MNI: -40, 8, 64, Z = 3.2), respectively. The activation differences between lures and foils were thresholded at Z > 2.3.

## Supplementary Tables

**Supplementary Table 1.** Behavioral results of endorsement rate and reaction time in the recognition test in young and older adults (mean ± standard deviation).

| Group | Proportion of “old” responses | | |  | Reaction Time | | | |
| --- | --- | --- | --- | --- | --- | --- | --- | --- |
|  | Target | Lure | Foil |  | TO | LO | LN | FN |
| Young adults | 0.90±0.09 | 0.59±0.18 | 0.12±0.09 |  | 1.27±0.18 | 1.49±0.19 | 1.65±0.26 | 1.44±0.23 |
| Older adults | 0.70±0.16 | 0.55±0.15 | 0.14±0.13 |  | 1.56±0.31 | 1.60±0.31 | 1.80±0.33 | 1.63±0.26 |

*Note*: TO: target judged as old, LO: lure judged as old, LN: lure judged as new, FN: foil judged as new. The unit of reaction time is second. Regarding the reaction time during retrieval, the mixed-design ANOVA revealed a significant interaction between age group and response type, *F*(3, 156) = 2.84, *p* = 0.04, a significant main effect of age group, *F*(1, 52) = 10.11, *p* = 0.002, and a significant main effect of response type, *F*(3, 156) = 29.78, *p* < 0.001. Independent sample *t*-tests revealed that older adults responded slower to recognize targets and reject foils than young adults (*ps* < 0.01), but there was no significant age difference in the reaction time to recognize lures (*p* = 0.12) and reject lures (*p* = 0.07). Paired-sample *t*-tests revealed that young adults took a longer time for false recognition than true recognition (*p* < 0.001), but no such difference was found in older adults (*p* = 0.19).

**Supplementary Table 2.** Behavioral results of the proportion and propensity for high-confidence recognition performance in young and older adults (mean ± standard deviation).

|  | Proportion of high-confidence recognition | |  | Propensity for high-confidence recognition | |
| --- | --- | --- | --- | --- | --- |
|  | Young adults | Older adults |  | Young adults | Older adults |
| Target | 0.81±0.14 | 0.59±0.21 |  | 0.66±0.08 | 0.55±0.09 |
| Lure | 0.38±0.14 | 0.42±0.18 |  | 0.30±0.07 | 0.38±0.05 |
| Foil | 0.04±0.04 | 0.10±0.12 |  | 0.03±0.03 | 0.08±0.07 |

*Note*: As conducted in previous studies (Dennis et al., 2007; 2008), we calculated the proportion of high-confidence recognition and the propensity for high-confidence recognition in young and older adults, separately. For example, among 108 words in the recognition test (i.e., 36 targets, 36 lures, and 36 foils), if a participant judged 30 targets, 15 lures, and 2 foils as “definitely old”, then the proportion of high-confidence false recognition would be 0.42 (i.e., 15/36 = 0.42), whereas the propensity for high-confidence false recognition would be 0.38 (i.e., 15/(30+15+2) = 0.38). The same logic was used to calculate these indices for targets and foils, respectively. In the current study, compared to young adults, older adults have lower proportion of high-confidence true recognition, *t*(52) = -4.51, *p* < 0.001, higher proportion of high-confidence foil recognition, *t*(52) = 2.50, *p* = 0.02. But there was no age group difference in the proportion of high-confidence false recognition, *t*(52) = 0.87, *p =* 0.39. To rule out the possibility that older adults had a higher individual bias to “definitely old” responses which led to higher propensity for high-confidence false recognition, we tested whether the overall “definitely old” responses differed between the two age groups. The result revealed that there was no age group difference in the overall “definitely old” responses (*t*(52) = 1.29, *p* = 0.21).

**Supplementary Table 3.** Regression analyses between behavioral performance and neural classification in each ROI.

| Predictors | True Recognition | | | | | | |  | False Recognition | | | | | | |
| --- | --- | --- | --- | --- | --- | --- | --- | --- | --- | --- | --- | --- | --- | --- | --- |
|  | Before controlling for activation | | |  | After controlling  for activation | | |  | Before controlling for activation | | |  | After controlling  for activation | | |
|  | *β* | *t* | *p* |  | *β* | *t* | *p* |  | *β* | *t* | *p* |  | *β* | *t* | *p* |
| Primary auditory cortex | | | | | | | | | | | | | | | |
| age | -0.04 | -0.19 | 0.85 |  | 0.01 | 0.03 | 0.98 |  | -0.19 | -0.60 | 0.55 |  | -0.10 | -0.33 | 0.74 |
| target-lure | -0.11 | -0.36 | 0.72 |  | -0.11 | -0.35 | 0.73 |  | -0.07 | -0.15 | 0.88 |  | -0.05 | -0.10 | 0.92 |
| target-foil | -0.07 | -0.27 | 0.79 |  | -0.07 | -0.25 | 0.80 |  | 0.07 | 0.18 | 0.86 |  | 0.07 | 0.18 | 0.86 |
| age × target-lure | 0.79 | 2.46 | **0.02** |  | 0.76 | 2.29 | **0.03** |  | 0.21 | 0.48 | 0.64 |  | 0.18 | 0.39 | 0.70 |
| age × target-foil | -0.50 | -1.83 | 0.07 |  | -0.57 | -1.98 | 0.05 |  | 0.18 | 0.48 | 0.64 |  | 0.06 | 0.14 | 0.89 |
| Planum temporale | | | | | | | | | | | | | | | |
| age | -0.18 | -0.94 | 0.35 |  | -0.16 | -0.81 | 0.42 |  | 0.05 | 0.18 | 0.85 |  | 0.15 | 0.59 | 0.55 |
| target-lure | -0.47 | -1.83 | 0.07 |  | -0.47 | -1.80 | 0.08 |  | -0.58 | -1.77 | 0.08 |  | -0.59 | -1.81 | 0.08 |
| target-foil | 0.11 | 0.47 | 0.64 |  | 0.13 | 0.51 | 0.61 |  | 0.36 | 1.14 | 0.26 |  | 0.41 | 1.24 | 0.22 |
| age × target-lure | 0.43 | 1.67 | 0.10 |  | 0.40 | 1.49 | 0.14 |  | 0.08 | 0.23 | 0.82 |  | -0.07 | -0.21 | 0.83 |
| age × target-foil | 0.12 | 0.51 | 0.61 |  | 0.11 | 0.45 | 0.66 |  | -0.13 | -0.40 | 0.69 |  | -0.19 | -0.61 | 0.55 |
| Medial occipital cortex | | | | | | | | | | | | | | | |
| age | 0.26 | 1.35 | 0.18 |  | 0.35 | 1.91 | 0.06 |  | -0.22 | -0.90 | 0.37 |  | -0.09 | -0.41 | 0.68 |
| target-lure | -0.13 | -0.45 | 0.66 |  | -0.22 | -0.76 | 0.45 |  | -0.49 | -1.34 | 0.19 |  | -0.58 | -1.63 | 0.11 |
| target-foil | 0.12 | 0.44 | 0.66 |  | 0.19 | 0.73 | 0.47 |  | -0.22 | -0.64 | 0.52 |  | -0.10 | -0.30 | 0.76 |
| age × target-lure | 0.12 | 0.42 | 0.67 |  | 0.11 | 0.39 | 0.70 |  | 0.44 | 1.19 | 0.24 |  | 0.43 | 1.24 | 0.22 |
| age × target-foil | -0.40 | -1.43 | 0.16 |  | -0.56 | -2.11 | **0.04** |  | 0.03 | 0.07 | 0.94 |  | -0.21 | -0.62 | 0.54 |
| Lateral occipital cortex | | | | | | | | | | | | | | | |
| age | 0.49 | 2.75 | **0.01** |  | 0.47 | 2.49 | **0.02** |  | 0.55 | 2.37 | **0.02** |  | 0.47 | 1.98 | **0.05** |
| target-lure | 0.35 | 1.18 | 0.24 |  | 0.30 | 0.97 | 0.34 |  | -0.12 | -0.32 | 0.75 |  | -0.14 | -0.36 | 0.72 |
| target-foil | 0.21 | 0.77 | 0.44 |  | 0.24 | 0.88 | 0.38 |  | 0.36 | 1.06 | 0.30 |  | 0.45 | 1.29 | 0.20 |
| age × target-lure | -0.54 | -1.82 | 0.07 |  | -0.52 | -1.70 | 0.10 |  | -0.96 | -2.52 | **0.02** |  | -0.85 | -2.18 | **0.03** |
| age × target-foil | -0.23 | -0.85 | 0.40 |  | -0.19 | -0.69 | 0.49 |  | -0.09 | -0.25 | 0.80 |  | -0.04 | -0.11 | 0.92 |

*Note.* In each regression model for each ROI, five regressors were modeled as predictors of behavioral performances (true recognition and false recognition), including age group (young adults = 1, older adults = -1), target-lure classification, target-foil classification, age × target-lure classification and age × target-foil classification. To control for the univariate activation level, the activation differences between targets and lures and between targets and foils in each ROI were included as nuisance variables. The regression results both before and after controlling for activation were listed in the table. Bold fonts represent significant results. The p-values presented in the table were uncorrected for multiple comparisons.

**Supplementary Table 4.** Brain regions showing neural discriminability above chance for each age group.

| Classification type | group | region | MNI coordinate | | | k | *t* |
| --- | --- | --- | --- | --- | --- | --- | --- |
|  |  |  | x | y | z |  |  |
| Target-Lure | YA | Right middle frontal gyrus | 38 | -2 | 58 | 31 | 4.67 |
|  |  | Right precentral gyrus | 26 | -12 | 62 | 44 | 4.57 |
|  |  | Left postcentral gyrus | -52 | -30 | 60 | 15 | 3.93 |
|  |  | Left supramarginal gyrus | -52 | -50 | 56 | 242 | 5.85 |
|  |  | Left dorsal lateral occipital cortex | -46 | -78 | 40 | 28 | 4.83 |
|  | OA | NA |  |  |  |  |  |
| Target-Foil | YA | left frontal pole | -40 | 58 | 8 | 767 | 4.58 |
|  |  | Left inferior frontal gyrus | -54 | 22 | 2 | 202 | 3.73 |
|  |  | Left middle frontal gyrus | -48 | 12 | 52 | 990 | 4.96 |
|  |  | Left superior frontal gyrus | -2 | 34 | 50 | 809 | 5.31 |
|  |  | Right superior frontal gyrus | 6 | 18 | 62 | 1040 | 4.06 |
|  |  | Left cingulate gyrus | -4 | -18 | 46 | 343 | 3.68 |
|  |  | Left precentral gyrus | -20 | -24 | 76 | 1720 | 6.55 |
|  |  | Right precentral gyrus | 26 | -26 | 72 | 1851 | 7.16 |
|  |  | Left postcentral gyrus | -66 | -20 | 22 | 1965 | 6.39 |
|  |  | Right postcentral gyrus | 38 | -26 | 64 | 1545 | 6.80 |
|  |  | Left supramarginal gyrus | -56 | -38 | 54 | 964 | 5.48 |
|  |  | Right supramarginal gyrus | 60 | -24 | 52 | 532 | 5.35 |
|  |  | Left angular gyrus | -46 | -56 | 54 | 237 | 4.36 |
|  |  | Right angular gyrus | 44 | -50 | 54 | 375 | 4.53 |
|  |  | Left superior parietal lobe | -46 | -44 | 62 | 1040 | 5.99 |
|  |  | Right superior parietal lobe | 18 | -56 | 70 | 972 | 4.34 |
|  |  | Left precuneus | -6 | -70 | 48 | 1492 | 5.56 |
|  |  | Right precuneus | 2 | -66 | 52 | 411 | 5.88 |
|  |  | Left inferior temporal gyrus | -60 | -56 | -12 | 238 | 4.38 |
|  |  | Right middle temporal gyrus | 54 | -56 | 8 | 134 | 4.03 |
|  |  | left middle temporal gyrus | -68 | -34 | -2 | 603 | 4.45 |
|  |  | Left heschls' gyrus | -54 | -16 | 4 | 73 | 3.41 |
|  |  | Left planum temporale | -54 | -32 | 14 | 209 | 4.37 |
|  |  | Left hippocampus | -30 | -38 | -6 | 89 | 3.63 |
|  |  | Left ventral lateral occipital cortex | -52 | -62 | 8 | 671 | 4.15 |
|  |  | Left dorsal lateral occipital cortex | -38 | -80 | 14 | 3292 | 4.36 |
|  |  | Right dorsal lateral occipital cortex | 44 | -58 | -34 | 1965 | 4.72 |
|  |  | Left lingual gyrus | -10 | -68 | -10 | 528 | 5.09 |
|  |  | Right lingual gyrus | 8 | -62 | -4 | 206 | 4.15 |
|  |  | Left occipital pole | -32 | -94 | -14 | 701 | 4.68 |
|  |  | Right occipital pole | 24 | -96 | 12 | 330 | 4.50 |
|  | OA | Left inferior frontal gyrus | -48 | 32 | -2 | 21 | 4.39 |
|  |  | left cingulate gyrus | -2 | 6 | 42 | 20 | 4.40 |
|  |  | Right paracingulate gyrus | 2 | 16 | 40 | 12 | 4.23 |
|  |  | right precentral gyrus | 44 | -18 | 66 | 258 | 4.72 |
|  |  | Left postcentral gyrus | -42 | -26 | 64 | 2689 | 5.15 |
|  |  | Right precuneus | 6 | -68 | 46 | 72 | 4.22 |
|  |  | Left middle temporal gyrus | -60 | -54 | -2 | 21 | 4.85 |
|  |  | Left ventral lateral occipital cortex | -50 | -72 | 0 | 10 | 4.17 |
|  |  | Right occipital pole | 2 | -90 | 20 | 204 | 4.54 |
|  |  | Left occipital pole | -30 | -96 | -12 | 26 | 4.19 |

*Note.* OA, older adults; YA, younger adults.

**Supplementary Table 5.** Regression analyses between left prefrontal activation for lures and target-lure classification accuracy in each ROI.

| Predictors | Before controlling for activation | | |  | After controlling for activation | | | |
| --- | --- | --- | --- | --- | --- | --- | --- | --- |
|  | *β* | *t* | *p* |  | *β* | *t* | *p* | |
| Primary auditory cortex | | | | | | | |  |
| age | 0.01 | 0.85 | 0.40 |  | 0.01 | 0.64 | 0.53 | |
| LPFC activation | 0.00 | -0.04 | 0.97 |  | -0.01 | -0.09 | 0.93 | |
| age × LPFC activation | 0.03 | 0.51 | 0.61 |  | 0.04 | 0.66 | 0.51 | |
| Planum temporale | | | | | | | |  |
| age | -0.01 | -0.37 | 0.71 |  | -0.01 | -0.50 | 0.62 | |
| LPFC activation | 0.02 | 0.24 | 0.81 |  | 0.02 | 0.26 | 0.79 | |
| age × LPFC activation | 0.04 | 0.53 | 0.60 |  | 0.05 | 0.64 | 0.52 | |
| Medial occipital cortex | | | | | | | |  |
| age | -0.02 | -1.37 | 0.18 |  | -0.02 | -1.21 | 0.23 | |
| LPFC activation | 0.05 | 0.81 | 0.42 |  | 0.05 | 0.83 | 0.41 | |
| age × LPFC activation | 0.10 | 1.62 | 0.11 |  | 0.09 | 1.50 | 0.14 | |
| Lateral occipitial cortex | | | | | | | |  |
| age | 0.05 | 3.19 | **<0.001** |  | 0.06 | 3.34 | **<0.001** | |
| LPFC activation | 0.11 | 1.87 | 0.07 |  | 0.12 | 2.08 | **0.04** | |
| age × LPFC activation | -0.18 | -3.20 | **<0.001** |  | -0.19 | -3.36 | **<0.001** | |

*Note.* In each regression model for each ROI, three regressors were modeled to predict target-lure classification accuracy, including age group (young adults = 1, older adults = -1), left prefrontal cortex (LPFC) activations for lures, and their interaction. To control for the univariate activations level, activation difference between targets and lures in each ROI was included in the model as a nuisance variable. The regression results both before and after controlling for activation were listed in the table. Bold fonts represent significant results. The p-values presented in the table were uncorrected for multiple comparisons.

**References**

Stark, C.E., and Squire, L.R. (2001). When zero is not zero: The problem of ambiguous baseline conditions in fMRI. *Proc. Natl. Acad. Sci. U. S. A.* 98(22)**,** 12760-12766. doi: 10.1073/pnas.221462998.
